# Longitudinal relationship between albuminuria in infancy and childhood

**DOI:** 10.1007/s00467-022-05850-5

**Published:** 2023-01-27

**Authors:** Valentina Gracchi, Sophie M. van den Belt, Eva Corpeleijn, Dick de Zeeuw, Hiddo J. L. Heerspink, Henkjan J. Verkade

**Affiliations:** 1 Department of Pediatrics, Beatrix Children’s Hospital, University of Groningen, University Medical Center Groningen, P.O. Box 30.001-CA13, Groningen, 9700RB The Netherlands; 2 Department of Clinical Pharmacy and Pharmacology, University of Groningen, University Medical Center Groningen, Groningen, The Netherlands; 3 Department of Epidemiology, University of Groningen, University Medical Center Groningen, Groningen, The Netherlands

**Keywords:** Albuminuria, Microalbuminuria, Albumin-creatinine ratio, Children, Epidemiology

## Abstract

**Background:**

Mildly increased albuminuria is common in the general adult population and is a strong predictor for cardiovascular events, even in otherwise healthy individuals. The underlying pathophysiological process could be endothelial dysfunction. Previously, we reported that increased albuminuria can also be found in 2-year-olds from the general population. We hypothesized that some individuals have constitutionally higher levels of albuminuria, possibly as an expression of early or inborn endothelial dysfunction. The aim of this study is to evaluate longitudinal persistence of albuminuria from infancy into school age.

**Methods:**

In the population-based GECKO (Groningen Expert Center for Kids with Obesity) cohort, urine was collected from 816 children at the age of 2 years as well as 12 years (random urine and first morning void urine, respectively). We evaluated prevalence and persistence of increased albuminuria (*U*_ACR_ ≥ 3 mg/mmol) at the two time points.

**Results:**

The prevalence of *U*_ACR_ ≥ 3 mg/mmol at 2 and 12 years of age was 31.9% (95% CI 28.7–35.2) and 3.1% (95% CI 2.0–4.5), respectively. *U*_ACR_ < 3 mg/mmol at both 2 and 12 years of age was present in 540 children (66.2%). Only 9 children (3.5%) of the 260 children with an *U*_ACR_ ≥ 3 mg/mmol at 2 years had an *U*_ACR_ ≥ 3 mg/mmol at 12 years (*p* < 0.001).

**Conclusion:**

Albuminuria in 2-year-olds does largely not persist until the age of 12, indicating that albuminuria at 2 years of age is not a marker for constitutional endothelial dysfunction in this cohort.

**Graphical abstract:**

**Figure:**
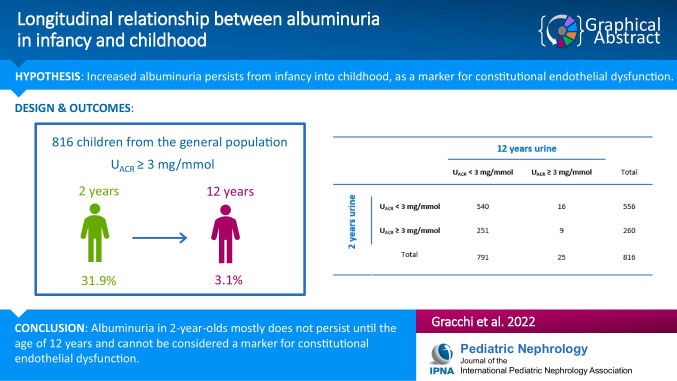
A higher resolution version of the Graphical abstract is available as Supplementary information

**Supplementary Information:**

The online version contains supplementary material available at 10.1007/s00467-022-05850-5.

## Introduction

Besides being a well-known marker for kidney disease, albuminuria is also an independent risk factor for kidney failure, cardiovascular morbidity, and all-cause mortality in adults [1–4]. Even a mild increase in albuminuria is a strong predictor for chronic kidney disease, cardiovascular events, and death, also in otherwise healthy individuals [5–8]. Mildly increased albuminuria is rather common in the general adult population, with a prevalence between 5.1 and 7.8% [6, 9, 10].

Previously, we reported that the prevalence of increased albuminuria (defined as urinary albumin concentration, *U*_AC_ ≥ 20 mg/L) was similar in Dutch toddlers and adults from the general population of the same geographical region [11]. The prevalence was 6.9% in 2-year-olds from the Groningen Expert Center for Kids with Obesity (GECKO)–Drenthe cohort (*n* = 1352) and 7.8% in adults from the Prevention of REnal and Vascular ENd-stage Disease study (PREVEND, *n* = 40,854), with a wide variation in albuminuria levels among individuals [11]. Based on these findings, we hypothesized that some individuals have constitutionally higher levels of albuminuria, possibly as an expression of inborn endothelial dysfunction, and that these individuals carry a higher kidney and cardiovascular risk throughout their lifetime. To test this hypothesis, long-term longitudinal data are needed, to determine if increased albuminuria persists longitudinally during childhood, and to ascertain if these individuals experience more kidney and cardiovascular events over time. Data on both of these research questions are still lacking.

In the present study, we addressed the first question and assessed whether increased albuminuria in 2-year-old toddlers from the general population longitudinally persisted until the age of 12 years.

## Methods

This study was nested in the GECKO–Drenthe cohort, an ongoing, population-based birth cohort with the primary goal of investigating prevalence and early risk factors for childhood overweight. All children born between April 2006 and April 2007 in the northern Dutch province of Drenthe were eligible. Detailed study design has been previously described elsewhere and is registered at www.birthcohorts.net [12]. Out of the 2842 children who had ever participated in the cohort, a random urine sample with *U*_AC_ measurement (nephelometric assay by Behring Nephelometer Analyzer II, Siemens; threshold 3.0 mg/L; urine collected using a pantyliner) was available from 1352 children at the age of 2 years, as previously described [11]. To correct albuminuria for urine dilution, we calculated urinary albumin-creatinine ratio (*U*_ACR_). Urinary creatinine concentration (*U*_CC_, enzymatic assay by Roche modular analyzer, Roche Diagnostics; threshold 0.1 mmol/L) and *U*_ACR_ were available from 1325 children at the age of 2 years (46.6% of the children in the cohort at 2 years).

At the age of 12 years, the 2299 children still actively participating in the cohort were asked to collect a first morning void urine sample at home, on a day with no symptoms of sickness. Of these 2299 children, 1311 children (57.0%) collected a first morning void urine sample between April 2018 and May 2019. Both *U*_AC_ (immunoturbidimetric assay by Cobas® 8000 c502 analyzer, Roche Diagnostics; threshold 3.0 mg/L) and *U*_CC_ (enzymatic assay by Cobas® 8000 c502 analyzer) were available in all 12-year samples, for a total of 1311 *U*_ACR_ measurements. Urine samples were also checked for hematuria and leukocyturia by urine dipstick. Urine samples with hematuria or samples collected during suspected viral or urinary tract infections were excluded.

Of the 1311 children with a *U*_ACR_ measurement at the age of 12 years, 816 children (62.2%) also had a *U*_ACR_ measurement at 2 years of age and were included for longitudinal comparison. Comparability of urinary albumin assessment methods at 2 and 12 years has been previously described [11]. Of these 816 children, 500 had a blood pressure measurement at 2 years. Inclusion flow chart and participant characteristics can be found in Supplementary Material (Fig. S1 and Table S1).

Comparison between albuminuria at 2 and 12 years was performed on the basis of the dichotomous variable *U*_ACR_ < 3 mg/mmol or ≥ 3 mg/mmol. Persistence of *U*_ACR_ ≥ 3 mg/mmol at 2 and 12 years was assessed using McNemar’s test, with a *p* value < 0.05 considered to be statistically significant. Associations between blood pressure at 2 years and *U*_ACR_ were analyzed by univariate linear regression analysis, using *U*_ACR_ both as continuous and dichotomous variable. *U*_ACR_ was log-transformed to account for skewed distribution. Data were analyzed using SPSS Statistics, version 28.

## Results

Of the 816 children included for longitudinal comparison, median *U*_ACR_ at 2 and 12 years was 1.9 mg/mmol (25–75th percentile: 1.1–3.7 mg/mmol; 95th percentile: 15.0 mg/mmol) and 0.4 mg/mmol (25–75th percentile: 0.3–0.6 mg/mmol; 95th percentile: 1.9 mg/mmol), respectively. The prevalence of increased albuminuria (*U*_ACR_ ≥ 3 mg/mmol) at 2 years was 31.9% (95% confidence interval [95% CI] 28.7–35.2) and at 12 years 3.1% (95% CI 2.0–4.5), as shown in Supplementary Material (Table S1).

The vast majority of the study participants had an *U*_ACR_ < 3 mg/mmol at both 2 and 12 years of age (*n* = 540; 66.2%). A smaller proportion of children had an *U*_ACR_ ≥ 3 mg/mmol at only one of the two time points, either at 2 years of age (*n* = 251; 30.8%) or at 12 years of age (*n* = 16; 2.0%). Of the 260 children with an *U*_ACR_ ≥ 3 mg/mmol at 2 years, only 9 children (3.5%, corresponding to 1.1% of the total number of children) had an *U*_ACR_ ≥ 3 mg/mmol also at 12 years (*p* < 0.001) (Table 1).

**Table 1:** Longitudinal assessment of increased urinary albumin-creatinine ratio, dichotomized in *U*_ACR_ < or ≥ 3 mg/mmol, in children from the general population at 2 and 12 years of age

		12-years urine sample		
		*U*_ACR_ < 3 mg/mmol	*U*_ACR_ ≥ 3 mg/mmol	Total
2-years urine sample	*U*_ACR_ < 3 mg/mmol	540	16	556
	*U*_ACR_ ≥ 3 mg/mmol	251	9	260
	Total	791	25	816

McNemar’s test: *p* < 0.001

Because *U*_ACR_ values are dependent on *U*_CC_, the results could be influenced by differences in muscle mass (and thus *U*_CC_) between 2 and 12 years of age. Therefore, we also compared albuminuria at 2 and 12 years on the basis of the dichotomous variable *U*_AC_ < 20 mg/L or *U*_AC_ ≥ 20 mg/L. The prevalence of increased albuminuria was more similar at the 2 time points: 6.7% at 2 years (95% CI 5.0–8.4) and 7.8% at 12 years (95% CI 6.0–9.6). Nevertheless, similar to the results obtained on the basis of *U*_ACR_, only a small proportion of children with increased albuminuria at 2 years still had increased albuminuria at 12 years (10.7%; data not shown).

At 2 years of age, there was no association between systolic blood pressure and *U*_ACR_ (analysis as continuous variable: coefficient 0.01, 95% CI − 1.06–1.18, *p* = 0.917; as dichotomous variable: coefficient 0.04, 95% CI − 1.39–3.39, *p* = 0.940), nor between diastolic blood pressure and *U*_ACR_ (continuous variable: coefficient 0.03, 95% CI − 1.41–0.64, *p* = 0.465; dichotomous variable: coefficient 0.43, 95% CI − 3.07–1.32, *p* = 0.112).

## Discussion

We investigated whether increased albuminuria generally persists in children between age 2 and 12 years, but this appeared not to be the case. Rather, increased albuminuria in early childhood is a predominantly transient phenomenon.

Our observations do not support the hypothesis that increased albuminuria in infants can be interpreted as an early marker of constitutional endothelial dysfunction in the general population. Theoretically, we cannot exclude that increased albuminuria could be a sign of constitutional endothelial dysfunction in the small subset of children with persisting increased albuminuria. Importantly, our present data do not exclude the possibility that constitutional endothelial dysfunction could become manifest as increased albuminuria only *later* in childhood or in young adulthood. In order to test this hypothesis, long-term longitudinal data on albuminuria from adolescence into adulthood, in combination with kidney and cardiovascular outcomes, would be needed. Preferably, these data should include repeated measurements of albuminuria at every time point and a large number of individuals.

The most important strength of our study is the 10-year follow-up of albuminuria in children from the general population, starting from a very young age. Another important strength is that 12-year-olds collected a first morning void urine sample, to avoid the measurement of orthostatic proteinuria, which is highly prevalent in school-aged children [13]. Finally, considerable efforts were made to address the issue of transient albuminuria during infections and to exclude children with possible kidney disease or menstruation (exclusion of children with hematuria).

We acknowledge as a limitation of our study that possible day-to-day variability of albuminuria could not be considered since only one sample per time point was available. Moreover, the number of children with urine at both time points is relatively small. We also acknowledge that the comparison between *U*_ACR_ at 2 and 12 years is complicated by the difference of *U*_CC_ in the 2 age groups, with lower *U*_CC_ in 2-year-olds and higher *U*_CC_ in 12-year-olds. This difference is due on one hand to the low muscle mass in toddlers (leading to low *U*_CC_ and, thus, to high levels of *U*_ACR_) and, on the other hand, to the first morning void collection at 12 years of age (leading to highly concentrated urine with high *U*_CC_ and, thus, to low levels of *U*_ACR_). Nevertheless, this consideration does not compromise the conclusion of our study that less than 5% of children with increased albuminuria at 2 years show persistent increased albuminuria 10 years later.

In conclusion, increased albuminuria in 2-year-old children mostly does not persist at the age of 12 years, indicating that albuminuria at 2 years of age is not a marker for constitutional endothelial dysfunction in this cohort.

## Supplementary Information

Below is the link to the electronic supplementary material.

Graphical Abstract (PPTX 74.1 KB)

Supplementary file1 (DOCX 30 KB)

## Data Availability

The datasets generated during and/or analyzed during the current study are available from the corresponding author on reasonable request.
