# Supplementary material for: Longitudinal relationship between albuminuria in infancy and childhood
Source: Pediatr Nephrol. 2023 Jan 27;38(8):2897–900. doi: 10.1007/s00467-022-05850-5 (PMC10393842; doi:10.1007/s00467-022-05850-5)
Supplement: Supplementary file 1 — Graphical Abstract (PPTX 74.1 KB) [file 467_2022_5850_MOESM1_ESM.pptx]

## Slide 1
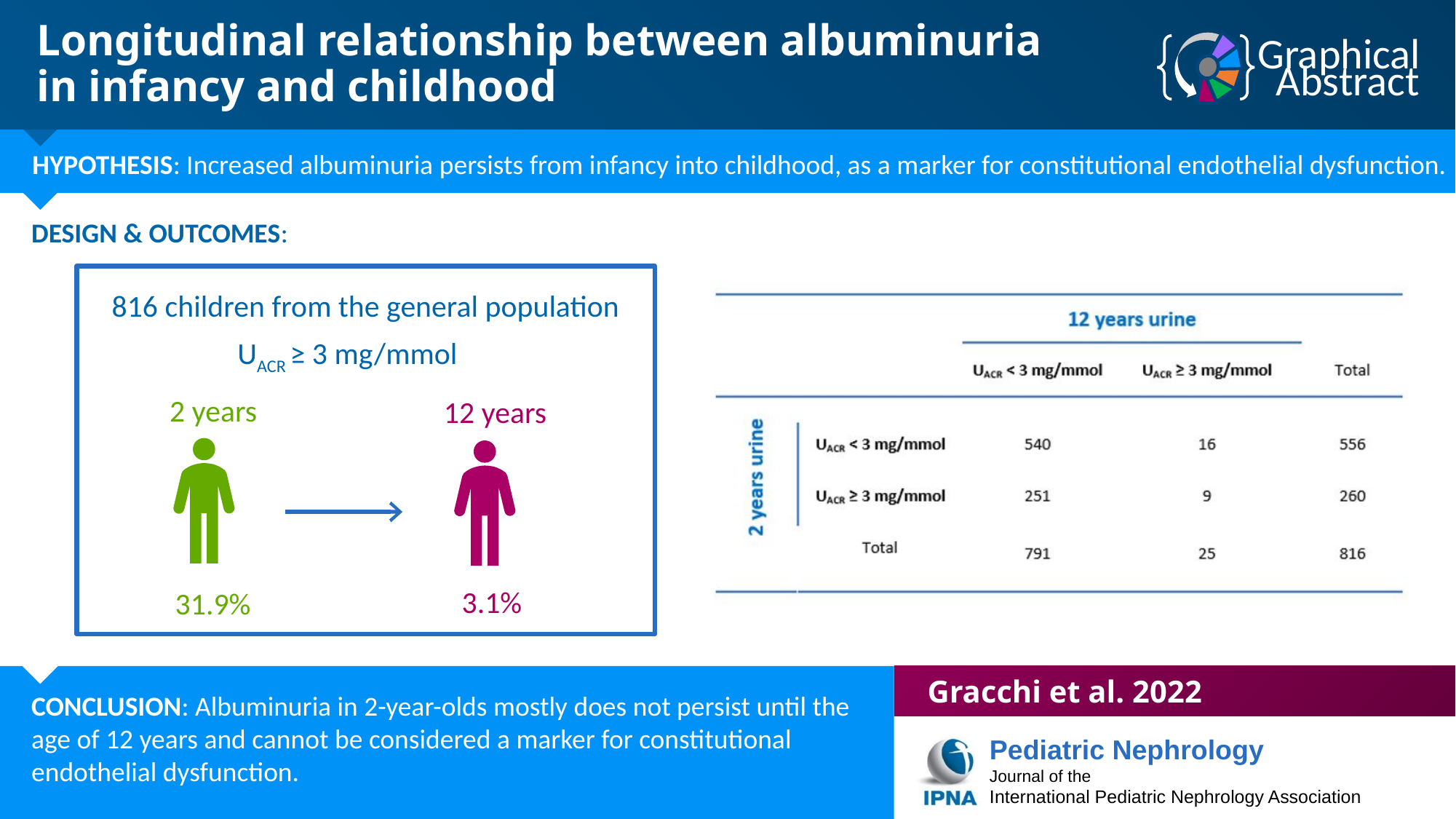

Longitudinal relationship between albuminuria
in infancy and childhood
HYPOTHESIS: Increased albuminuria persists from infancy into childhood, as a marker for constitutional endothelial dysfunction.
DESIGN & OUTCOMES:
816 children from the general population
UACR ≥ 3 mg/mmol
2 years
12 years
3.1%
31.9%
Gracchi et al. 2022
CONCLUSION: Albuminuria in 2-year-olds mostly does not persist until the age of 12 years and cannot be considered a marker for constitutional endothelial dysfunction.
