# Supplementary material for: Longitudinal relationship between albuminuria in infancy and childhood
Source: Pediatr Nephrol. 2023 Jan 27;38(8):2897–900. doi: 10.1007/s00467-022-05850-5 (PMC10393842; doi:10.1007/s00467-022-05850-5)
Supplement: Supplementary file 2 — Supplementary file1 (DOCX 30 KB) [file 467_2022_5850_MOESM2_ESM.docx]

**Supplementary Material Figure S1** Flowchart of inclusion

Withdrawal informed consent, n = 47

No data/essential data missing, n = 108

Eligible children, n = 5326

Not willing to participate /no informed consent, n = 2329

Children with a urine albumin-creatinine ratio measurement available at both 2 and 12 years, n = 816

Withdrawal informed consent/ lost to follow-up, n = 543

Not willing to participate or not succeeded in urine collection at 2 years, n = 947

No urine creatinine concentration available at 2 years, n = 27

Informed consent, n = 2997

Urine collection at 2 years, n = 1352

Urine albumin-creatinine ratio measurement available at 2 years, n = 1325

Ever actively participated, n = 2842

Withdrawal informed consent / lost to follow-up/ no response to invitation for 12 years urine collection or invitation declined, n = 496

Incomplete informed consent form for 12 years urine collection, n = 5

Incorrect urine sample collection, hematuria, suspected viral or urinary tract infection, n = 8

**Supplementary Material Table S1** Characteristics of the study population

|  | **Children with U_ACR_**  **at 2 and 12 years**  **(n= 816)** | |
| --- | --- | --- |
|  | **Data at 2 years** | **Data at 12 years** |
| Age, years (25th-75th perc) | 2.1 (2.1 – 2.3) | 11.7 (11.8 - 12.0) |
| U_AC_, mg/L (25th-75th perc) | 3.0* (3.0* - 7.1) | 4.5 (3.0* - 8.7) |
| U_AC_ ≥ 20 mg/L, n (%) | 55 (6.7) | 64 (7.8) |
| U_CC_, mmol/L (25th-75th perc)  U_ACR_, mg/mmol (25th-75th perc)  U_ACR_ ≥ 3 mg/mmol, n (%)  Length, cm (SD) | 2.7 (1.3 – 4.3)  1.9 (1.1 – 3.7)  260 (31.9)  89.7 (3.4) | 12.7 (9.5 – 16.2)  0.4 (0.3 – 0.6)  25 (3.1)  148.0 (6.7) |
| BMI, kg/m2 (25th-75th perc) | 16.3 (15.4 – 17.2) | 17.1 (16.0 – 18.8) |
| Waist circumference, cm (25th-75th perc) | n.a. | 62.0 (59.0 – 66.7) |
| Systolic BP, mmHg (SD) | 100.6 (12.7) | 108.2 (9.6) |
| Diastolic BP, mmHg (SD) | 59.8 (11.6) | 63.5 (7.2) |
| Male sex, n (%) | 417 (51.1) | |
| Gestational age, weeks (25th-75th perc) | 40.0 (39.0 – 41.0) | |
| Birth weight, g (SD) | 3562 (571) | |

Values for continuous variables are reported as mean (standard deviation) or median (25th–75th percentile), as appropriate; values for categorical variables as number (percentage). U_AC_, Urinary Albumin Concentration (*3 mg/L is the lower limit of detection); U_CC_, Urinary Creatinin Concentration; U_ACR_, Urinary Albumin Creatinin Ratio; BMI, Body Mass Index; BP, blood pressure; n.a. not available. BP at 2 years available in n=500 children, at 12 years in n=694 children
